# 

**Published:** 1893-04

**Authors:** 


ILLINOIS STATE DENTAL SOCIETY AND IOWA STATE DENTAL SOCIETY—JOINT MEETING.—The twenty-ninth annual meeting of the Illinois State Dental Society will be held at Rock Island, May 9-12, inclusive, The thirtieth annual meeting of the Iowa State Dental Society will be held at Davenport, May 9 12, inclusive. These cities are located on opposite sides of the Mississippi river and arrangements will be made to hold the meeting jointly, so that those in attendance at the meeting ef either society will have an opportunity to listen to the papers, take part in the discussions and witness the clinics of both societies. No efforts will be spared to make this union meeting one of the most interesting in the history of each society. Members of both societies are urgently requested to attend. All dentists are cordially invited to be present. Every one should bring models, specimens, appliances or anything that may be of interest to the profession.

LOUIS OTTOFY, Secretary Illinois State Dental Society, Chicago.

W. O. KULP, Chairman Executive Committee Iowa State Dental Society, Davenport, Iowa.

